# *In vitro* Evaluation of Performance Properties of Sponge Hemostatic Dressings (Review)

**DOI:** 10.17691/stm2020.12.1.16

**Published:** 2020

**Authors:** D.A. Severinov, S.V. Lazarenko, K.A. Sotnikov, V.V. Pohozhay, P.V. Ansimova, V.A. Lipatov

**Affiliations:** Assistant, Department of Human Anatomy, Kursk State Medical University, 3 K. Marx St., Kursk, 305041, Russia; Assistant, Department of Oncology, Kursk State Medical University, 3 K. Marx St., Kursk, 305041, Russia; Radiologist, Computer Tomography Unit, Department of Radiodiagnostics, S.P. Botkin City Clinical Hospital, Moscow Department of Public Health, 5, 2-y Botkinskiy Proezd, Moscow, 125284, Russia; Associate Professor, Department of Oncology, Gomel State Medical University, 5 Lange St., Gomel, 246000, Republic of Belarus; Student, Kursk State Medical University, 3 K. Marx St., Kursk, 305041, Russia; Associate Professor, Professor, Department of Operative Surgery and Topographic Anatomy, Kursk State Medical University, 3 K. Marx St., Kursk, 305041, Russia

**Keywords:** hemostatic implants, hemostatic dressings, parenchymal bleeding, properties of implants, implant testing *in vitro.*

## Abstract

Dressings for restoring organ defects and/or hemostasis in the injury site are being actively applied in operational units. These dressings are used in various surgeries and are widely represented in the foreign and domestic markets of medical products. Many local implants have different levels of hemostatic activity, which requires standardization of the algorithm of choice and the methods of their study.

Here the methods of studying the performance properties of hemostatic implants *in vitro* have been considered and evaluation criteria of their physical, chemical and organoleptic properties *in vitro* have been proposed. This will allow a researcher to choose optimal variants of samples for further experiments on biological models more effectively as well as to save funds, time and reduce the number of experiments *in vivo*.

## Introduction

Abdominal surgery today shows a tendency to organ-preserving operative treatment of parenchymal organs (kidneys, liver, spleen) injuries due to their high functional significance [1, 2]. Traditional hemostatic methods such as U-shaped sutures, swabbing with an omentum, etc. are still as topical as ever, because they provide compression of intra-organic blood vessels. However, when there is capillary, superficial bleeding it is reasonable to use alternative sparing methods as suturing involves additional tissue damage. It justifies rationality of using non-suture technologies for replacing organ defects and/or hemostasis in the injury area [3, 4].

There are several methods provided both by foreign and national manufacturers. Their effectiveness depends on the approach to the production of morphological basis of samples, the basis usually being a sponge structure of animal (collagen) or synthetic (cellulose salts) origin [5]. This sponge structure has adhesive properties; therefore the implant surface is fixed to the surface of the injured organ without any supplementary suture material and other means. High adhesion characteristics are an indispensable criterion to stop bleeding [6–10]. Apart from adhesive properties, there are some other important factors, such as absorbability and sorption, which depend on the chemical structure and spatial structure organization (porosity of hemostatic sponges) [11].

Despite the increase in the number of surgeries and a growing need of surgical units in hemostatic implants used for local hemostasis, the common algorithm for evaluating effectiveness of these materials has not been established yet.

We proposed [12] an algorithm of choice of hemostatic means applied in surgeries mainly on parenchymal abdominal organs. According to this algorithm, step I is a screening investigation of physical-mechanical properties of implants with *in vitro* methods; step II is a method of an “acute” *in vivo* experiment which is necessary for identifying the duration of bleeding and the volume of blood loss; step III is a method of “chronic” *in vivo* experiment — investigation of intensity of reaction of animal tissues to the means of subcutaneous implantation and in an injury model of parenchymal organs, etc. These steps allow for a comprehensive study of hemostatic capacity of sponge hemostatic dressings and elimination of possible negative effects of their application.

The majority of modern researchers compare hemostatic means based on the findings obtained in experiments *in vivo* [13]. However, in some cases, the experiments on animals could be avoided. In this work, we consider performance properties of implants that can be evaluated in studies *in vitro*.

By the term “performance characteristics of medical products” we understand a set of properties that reveal a possibility of applying this medical product and using it effectively [14]. For the purpose of a more comprehensive analysis we divided the performance properties into the following groups (according to the methods of identification): chemical, physical, organoleptic (see the Table).

**Table:** Criteria of evaluation of performance properties of sponge hemostatic dressings

Physical properties		Chemical properties		Organoleptic properties
Mechanical parameters	Sorption parameters	Qualitative parameters	Quantitative parameters	
Complete, residual deformity Elasticity Nominal strength at break Percentage elongation at break Hardness Plasticity	Linear and surface density of tissues Adhesion degree Capillarity Sorption capacity and centrifugation Actual amount of water Water content	Reaction of water extract Reaction to identification of presence of the stated substances Reaction to identification of presence of ballast substances	Identification of quantity of the stated substances Identification of quantity of ballast substances	Color Density Adhesion to gloves Modelability

Currently, only a part of the methods provided below is regulated by the corresponding documents (GOST), represented in the State Pharmacopeia, etc. These methods have not changed from the moment of their development, but they are still relevant especially when used from a comprehensive perspective.

## Identification of physical properties

Investigation of performance characteristics of hemostatic implants should be started with the evaluation of ***sorption parameters*.** These parameters characterize the underlying property of dressings — adhesion [15, 16]. According to the physical understanding, adhesion is conjunction of surface layers of two heterogeneous bodies (phases) that are in contact. For a better understanding it is relevant to have a look at the laws of classical mechanics, to be more exact to the third Newton’s law which reads: “For every action, there is an equal and opposite reaction”. In other words, the effort to separate surfaces is equal to the strength of their interaction (adhesion): F_1_=–F_2_. Evaluation of the adhesion force is the most important for the development of the technology to change the product properties and enhance its effectiveness [17].

The study of adhesion is a complicated multi-sided multi-step process when one takes into consideration not only the properties of contact surfaces (texture, density, elasticity, water affinity, viscosity, etc.), but also their contact environment, in particular, humidity [18, 19].

Investigation of these characteristics can be done with calculation methods based on such key parameters as length, width, and weight of samples. It does not require any expensive and hard-to-access equipment.

During the analysis linear and surface density is identified [20–22]. The linear density of the sample *М_l_* (g/cm) is calculated according to the formula:

$$M_{l}=m_{l}\cdot 10^{2}/L,$$

where *m_l_* is the weight of the point sample (g); *L* is the average length of the point sample (cm).

The surface density *M_s_* (g/cm) is calculated according to the following formula:

$$M_{s\;}=\frac{m_{l}\cdot 10^{4}}{L\cdot b},$$

where *b* is the average width of the point sample (cm); *m_l_* is the weight of the point sample (g); *L* is the average length of the point sample (cm).

Some authors [23–25] suggest identifying the apparent specific weight of the implant according to the formula:

$$d_{app}=P/V.$$

And the formula of volume (*V*) of through pores and one-side open pores is as follows:

$$V=\left(m_{2}–m_{1}\right)/d_{xyl},$$

where *m*_1_ is the weight of air-dry sample of 2×2×1 cm size; *m*_2_ is the weight of the sample after it has been soaked in the solvent medium (for example, in xylol, specific weight (*d_xyl_*) is which is equal to 0.8812 g/cm^3^) [26].

The total sponge porosity (*P*) is calculated according to the formula:

$$P=V_{1}/V_{2}\cdot 100\%,$$

where *V*_1_ is the volume of air-dry sample (4 cm^3^); *V*_2_ is the volume after soaking in the solvent medium.

An important step of testing is identifying an ability of samples to absorb moisture [27, 28]. Open porosity (*Р_open_*), i.e. the volume of pores contacting with the environment is calculated as follows:

$$P_{open}=\left(m_{2}–m_{1}\right)/m_{1}\cdot 100\%,$$

where *m*_1_ is the weight of the initial sample with the size of 2×2×1 cm; *m*_2_ — is the weight of the sample after it has been soaked in water for 4 h.

To identify the moisture content of samples a group of researchers [29–31] proposes the following method: ten weighed portions with the square area of 1×1 cm weighing 5 g each are dried at the temperature of 50–250°С. In 1 h 30 min they are weighed for the first time. Then the samples are cooled to 36°С and are placed into a drying chamber for 30 min. Then they are cooled again and weight check is performed. The actual humidity (*W_act_*) is calculated according to the formula:

$$W_{act}=(1–\frac{Z_{2}}{Z_{1}})100\%,$$

where *Z*_1_ is the weight before drying; *Z*_2_ is the weight after the second drying. The arithmetic mean is taken for the result.

To identify a sorbing capacity of implants the following methods are used [32, 33]. The traditional method of measuring a sorbing capacity is based on weighing dressings before and after soaking them in liquid. However, it can give only information about mechanical filling of the system of capillaries and pores of the studied sample.

There are several variants of doing this investigation [34–36]. For instance, three weighed samples are placed on the bottom of three funnels covered with stops. The funnels are filled with water to the top. In 10 min the stops are removed. When the water streams down (2–3 min) the samples are turned to the other side for 10 min to remove the excess liquid. Then they are weighed and the actual amount of water (*К_act_*), sorbed by each sample is identified as follows:

$${К}_{act}=n\cdot 100/m,$$

where *n* is the weight of water sorbed by the sample (g); *m* is the weight of the sample (g). The arithmetic mean of three samples is the sponge sorbing capacity.

Alam et al. [37] suggest keeping weighed samples of the tested materials with the mass of 0.05–0.09 g in whole blood. The percentage of the weight gain is considered to be a sorbing capacity.

In study [38] after the above-mentioned procedures have been carried out, it is recommended to centrifuge the samples for 45 min at 6000 rpm, and the sorbing capacity should be identified by the difference in sample weights before sorption and after centrifuging.

The sorbing capacity of dressings can be studied with the methods of evaluation of capillarity — by increasing or decreasing the level of liquid in capillaries (narrow tubes, free-form canals, porous bodies). During the investigation one measures the rate of solution rise in a glass tube densely filled with samples in a specific way. For this purpose samples with the same weight are shaped into bands. A glass tube is densely filled with these bands from the zero mark. Then this tube is placed into a vessel filled with colored water in such a way that the liquid is at the marked level. The liquid height (*h*) is measured in 10 min from the moment of contact of the colored liquid and the zero mark. For *h* the highest point of the contour of the soaked dressing is taken. Capillarity (*К*, mm) is calculated according to the formula *К*=*h*/10.

Higher inaccuracy of this method should be mentioned as most findings are obtained by the researcher visually without any testing and measuring devices [39, 40].

Legonkova et al. [29–31] suggest evaluating performance characteristics of dressings according to the following parameters:

swelling ratio (g): *Q=(M_w_–M_d_)*/*M_d_*, where *М_w_* and *М_d_* are weights of wet and dry samples at 25°С, respectively;

constant of swelling rate (min^–1^): *K*(*t*)=In*Q_m_*/(*Q_m_*–*Q*), where *Q* is the amount of liquid sorbed by 1 g of substance for the time *t*, *Q_m_* is the maximum amount of the sorbed liquid (highest swelling);

apparent density for porous sponges (g/cm^3^): ρ*_app_*=*m*/*V*.

Considering instrumental methods of evaluating physical parameters it is worth mentioning that authors [41–44] suggest studying the specific square area of the surface (including this of dressing materials) with NOVA 2200 analyzer (Quantachrome Corp., USA) using nitrogen as an adsorbing gas. The specific square area of the surface is calculated with Brunauer–Emmet–Teller equation which includes two stages: identification of the monolayer capacity by adsorption isotherm and calculation of the specific surface area using the molecular square area of gas. Here the following assumptions are possible: the surface of the adsorbent is homogeneous; adsorbent-adsorbate interaction is stronger than adsorbate-adsorbent interaction; interaction of adsorbed molecules is taken into account only in the direction which perpendicular to the surface and is regarded as condensation. The surface square area of adsorbent is defined by the gas volume related to the monomolecular layer and the square area of the cross-section of the molecule of the adsorbed gas.

To evaluate the adhesion degree, the character of micro-relief and electrical conductivity of samples it is possible to use atomic-powered spectroscopy which principle of action is based on the force interaction of the membrane surface and the scanning probe. Force adhesion is measured by the square area of the sensor contact with the surface of the tested sample in different points. Hence, the “advance/retract” line graphs (contact–no contact) are obtained. Information about the degree of electrical conductivity is obtained with current spectroscopy. The character of relief is identified by the mean deviation of the sample surface from the isoline in 100 randomly selected points.

The investigation of polymer membranous implants *in vitro* with the above-mentioned methods stated [45] that the highest parameters of adhesion force are typical of samples with high values of surface roughness parameters, while the lowest adhesion values are observed in samples with smooth (flatter) surface.

Properties of hemostatic dressings are studied with ***mechanical methods*** to characterize such property as elasticity [46, 47]. It is of practical interest for surgeons as the convenience of using implants intraoperatively and/ or in the wound depends on it. Mechanical properties are evaluated with such a device as a tearing/compressing/smashing machine (the device accuracy must meet GOST 28840 requirements [48]). These machines operate according to the principle of transformation of kinetic energy produced by the servomotor into the load force applied to the tested sample [48–50].

The tested sample must be rectangular in its cross-section, have no superficial film and visible defects. Two lines marking the working area are applied on the samples with a marker with parallel plates. The internal distance between the lines must be 25–50 mm with error margin of ±1% [51, 52].

Furthermore, it is recommended to model samples with a die cutter used for cutting details and rubber blanks.

After the sample has been prepared according to the regulatory acts and conditions of the experiment it is fixed into the clams of the machine [53]. The clamps are tightened to provide a symmetrical position of the sample for even distribution of the occurred tension along the square area of its cross-section and also to prevent shifts of the sample during the experiment and its damage at the place of fixing. The distance between the clamps of the testing machine corresponds to the minimally admissible length of medical products. It often determines the sample size for testing [54–57]. Sometimes the person conducting an experiment has to modify the clamp with supplementary original constructions.

Tests on preliminary indention are carried out in the following way. A sample is placed on the bearing surface of the machine in such a way that its center is under the indenter center. The samples that have slots on one side must be located in such a way that this side is near the bearing surface [58–60]. Load is applied to the tested surface and the indenter is loaded into the sample with the rate of 100±20 mm/min till deformity of 70±2.5% from the initial thickness is reached, then the load is released at the same rate. In case the parameter of hardness I identified after load release the indenter is loaded into the sample for 40±1% from the initial height of the material for 30 s [61].

The remained deformation of compression is defined by the difference between the initial and end thickness of the tested object. The end thickness is the value obtained from the sample compression for the given period of time (72 h), at the specific temperature mode (20±2°С), humidity (65±5%) and the time of reconstitution of the initial thickness. When the load is removed, changes in the sample thickness are identified [62–64].

Before the elasticity test the central part of the sample is marked to limit the length of the test surface which must be at least 50 mm for rectangular samples in accordance with GOST 29104.1-91 [20, 65–67].

The nominal strength and percentage elongation at break are set with the method of stretching with constant load. The thickness of the object is measured at five evenly located or at two random points in sample area. The thickness of test samples should not differ by more than ±2% [68–70].

Then parameters of elasticity and plasticity are calculated. Elastic modulus (Young’s modulus) characterizes an ability of material to resist deformity [71, 72]. It is identified according to the formula *Е*=σ/ε, where σ is tension; ε is elongation at break.

By plasticity we understand an ability of material to change shape and sizes without being destroyed through exposure to external force. Plasticity is measured by such parameters as percentage elongation (contraction) δ and lateral contraction (expansion) ψ during a static test for stretching (contraction) [73–75]:

$$\delta =\left(l_{k}–l_{0}\right)/l_{0}\cdot 100\%,$$

$$\psi =\left(F_{k}–F_{0}\right)/F_{0}\cdot 100\%,$$

where *l*_0_ and *l_k_* are the lengths of the sample before and after break, *F*_0_ and *F_k_* are the square area of cross-section of the sample before and after destruction.

The conclusion about material plasticity is made according to the correlation of the obtained findings with the following criteria: flexible — δ>5%, fragile — δ<5%. However, ideally, these criteria in physics and mechanics of materials are applicable only to solid substances and bodies (metals, wood, etc.) [76–78].

## Identification of chemical properties

Chemical methods of evaluating properties of sponge hemostatic dressings can be divided into ***qualitative*** (identification of presence of substances with the given level of purification stated by manufacturer) and ***quantitative*** (their percentage correlation, etc.). It is significant to study traces of substances (including their toxically significant amount) used in the technological process — reagents that must not be present in the finished product. Improper storage conditions or quality of reagents can considerably affect the end properties of implants, their effectiveness potentiate tissue reaction of the macroorganism and therefore reduce biological inactivity of the material. The present of “extra” (“ballast”) substances can lead to the development of paradox allergic reactions, or formation of teratogen and oncogene properties of hemostatic dressings [79, 80].

One of the simplest and the most available and representative from the practical point of view methods is the method of identification of water extract reaction [81–83]. The studied material is boiled in distilled water for 15 min and cooled. The reaction of the obtained solution is studied with an indicator [84, 85]. This method allows evaluating pH of the sample medium and anticipating the course and the result of biochemical reactions occurring in the macroorganism after implant introduction.

## Identification of organoleptic properties

Organoleptic methods deserve special attention. They are universal and available. Physical and chemical properties of sponge hemostatic dressings are identified instrumentally or by calculation, while organoleptic (manipulation) properties should be identified by invited experts (expert method), i.e. practicing surgeons [86–88]. Evaluation is performed by comparison of experimental groups by giving those scores or ratings. Subjective parameters (such as sample color before and after implantation into the wound, density, adhesion to gloves, modelability, etc.) can be used as criteria. Each of the given criteria is significant as it determines the way surgery goes, convenience of surgical manipulations and sometimes their duration [89–91]. Organoleptic methods of investigation have an integral character and can replace long multi-factor evaluation of materials which requires expensive equipment [92–94].

## Conclusion

Development and investigation of sponge hemostatic dressings is performed in different directions. Various approaches to modification of dressings are being developed to potentiate their effectiveness [95–100]. It, in its turn, requires the development of screening (mass) study of effectiveness of these materials which will allow making an early comparative analysis of various samples. In our opinion, screening tests may be performed with *in vitro* methods. However, this evaluation will be incomplete due to considerable inaccuracies compared to *in vivo* experiments as a macroorganism is a complex open system with multiple variables that cannot be taken into consideration today even by high-capacity computing equipment.

The algorithm suggested by us [12] and the provided methods of evaluation of performance properties of hemostatic properties *in vitro* will help a researcher to understand the sample choice at the initial stages of work. Algorithmization will give general understanding of mechanisms of action of hemostatic dressings which can be further used in comparison of their effectiveness and finally will alleviate the choice of prospective tested samples from the wide range of hemostatic products.
